# Assessing Methane Emissions from Shale Gas Production in China: A Two-Tiered Mobile Measurement Approach

**DOI:** 10.1021/acs.est.5c01953

**Published:** 2025-12-23

**Authors:** Pu Hong, Yuzhong Zhang, Wenrui Shi, Shuang Zhao, Xin Feng, Minghao Zhuang, Xi Lu, Meiyu Guo

**Affiliations:** † Department of Geography, 26679 Hong Kong Baptist University, Kowloon Tong, Hong Kong SAR 999077, China; ‡ Key Laboratory of Coastal Environment and Resources of Zhejiang Province, School of Engineering, 557712 Westlake University, Hangzhou, Zhejiang 310024, China; § College of Resources and Environmental Sciences, National Academy of Agriculture Green Development, Key Laboratory of Plant-Soil Interactions, Ministry of Education, 34752 China Agricultural University, Beijing 100193, China; ∥ State Key Laboratory of Regional Environment and Sustainability, School of Environment, Tsinghua University, Beijing 100084, China; ⊥ Institute of Advanced Technology, Westlake Institute for Advanced Study, Hangzhou, Zhejiang 310024, China; # China Institute for Carbon Neutrality, Tsinghua University, Beijing 100084, China; ¶ Beijing Laboratory of Environmental Frontier Technologies, Tsinghua University, Beijing, 100084, China

**Keywords:** shale gas, methane emission, point source Gaussian method, STILT

## Abstract

China, holding the world’s largest shale gas reserves, lacks precise data on methane emissions from its rapidly expanding production. We introduce a two-tiered mobile measurement approach, using a mobile laboratory to measure methane concentrations across 125 well pads (approximately 750 wells) distributed among four major production blocks (Changning, Weiyuan, Fuling, and Luzhou). These blocks contributed 84% of China’s total shale gas production in 2023, providing the first comprehensive ground-level measurements. Stationary downwind monitoring of well pads revealed emission rates from 0.002 to 98.86 kg/h, validated through mobile observations of methane concentrations across the region. Notably, emissions were highly concentrated, with 89% originating from just 10% of the well pads. For 2023, the extrapolated methane emissions from China’s shale gas production were estimated at 16,842 t (6,444–29,991 t, 95% CI), corresponding to a methane leakage rate of 0.10% (0.04%–0.17%, 95% CI). This rate is lower than major U.S. fields and similar to that of U.S. dry gas fields. Our research identifies gas lift venting, incomplete combustion from compressors, and process venting as significant sources of super-emissions in China’s shale gas upstream production chain. The methodology employed, based on comprehensive and targeted field measurements, demonstrates its effectiveness in providing a scientific basis for formulating precise and effective regulatory policies on methane emissions.

## Graphical abstract

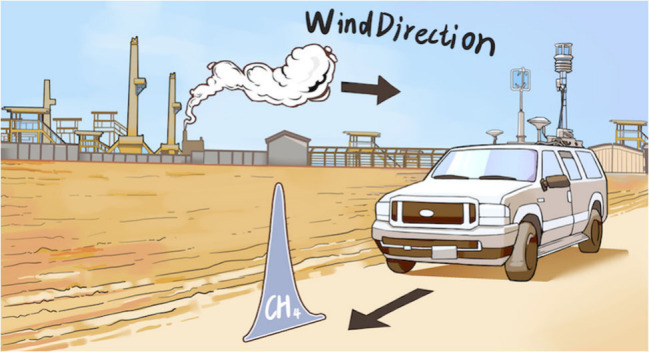

## Introduction

Shale gas is widely recognized as a transitional energy source that helps the shift from coal to renewable energy, primarily due to its lower carbon emissions.^1^ However, its principal constituent, methane, is the second most significant greenhouse gas following CO_2_ and has been responsible for 0.6 °C of global warming since preindustrial times.^2^ Methane possesses a global warming potential (GWP) approximately 84 times that of CO_2_ on a 20-year time scale,^3,4^ and mitigating methane emissions from shale gas production is crucial for climate change mitigation. The hydraulic fracturing in shale gas development can result in additional methane emissions compared with conventional gas extraction,^5,6^ and maintaining a leakage rate below 2.4%–3.2% on a 20-year scale is critical for considering shale gas as an effective energy source.^1,6,7^ However, shortcomings persist in methane monitoring efforts within the shale gas industry. These include high uncertainty in survey results, data gaps in certain regions, a lack of time series data, and poor data consistency within the same area, all of which significantly impede the establishment and tracking of emission reduction targets.

China holds the world’s largest technically recoverable reserve of shale gas at 31.6 trillion cubic meters^8^ and has experienced rapid growth in its shale gas industry, with annual production increasing from only 0.2 billion cubic meters (bcm) in 2013 to 25 bcm in 2023, making it the world’s third-largest shale gas producer.^9,10^ Shale gas production is integral to China’s 14th Five-Year Plan (2021–2025) and is aligned with the government’s carbon peak and neutrality action plan, positioning it as a crucial element in the nation’s shift toward a sustainable energy future.^11,12^ However, due to the lack of relevant regulations and policies, empirical data on methane emissions from China’s upstream shale gas industry are nearly nonexistent, with most quantitative studies concentrated in the United States and Canada. Studies have revealed significant methane emissions from shale gas production in the U.S., with leakage rates ranging from 0.03% to 1.9%.^7,13–15^ Notably, the top 10% of emitters were found to contribute 77% of the total emissions,^13^ highlighting the importance of identifying and addressing the emissions from these major contributors to effectively mitigate methane emissions. While Xue et al.^16^ attempted to quantify methane emissions from shale gas production in the Sichuan Basin, their limited sample size of only three well pads is insufficient for robust understanding of overall emissions from China’s shale gas industry. Despite China’s commitment to emission reduction,^17^ the absence of specific targets has resulted in limited industry motivation for reduction measures, necessitating in-depth sector-specific investigations to guide the establishment of emission reduction targets.

Methane emission monitoring in the oil and gas industry traditionally relies on either Bottom-Up or Top-Down approaches, each with distinct advantages and limitations. Bottom-Up methods, utilizing ground-based monitoring equipment to enable precise emission source identification and quantification, facilitate the identification of methane emission mechanisms, and improve existing emission factors. However, these methods are constrained by temporal and spatial limitations; extrapolation to regional emission estimates often carries potential errors due to sampling bias.^18,19^ Furthermore, their substantial investment in personnel and equipment results in high costs and long timeframes. Top-Down approaches have emerged as a complementary methodology, with numerous studies leveraging satellite remote sensing and aerial monitoring technologies to assess regional-level methane emissions.^20–22^ These methods enable efficient and continuous acquisition of emission data across large geographic areas, providing independent verification of Bottom-Up emission inventories.^23,24^

During an investigation of shale gas production regions in China’s Sichuan Basin, quantification of regional methane emissions relied solely on ground-level monitoring with extrapolation due to the absence of high-quality satellite data and unattained authorization for drone operations. To overcome these data limitations, we developed a novel two-tier methodology: Tier 1 employs the conventional OTM-33A to quantify emissions from well pads within target regions, while Tier 2 utilizes the Stochastic Time-Inverted Lagrangian Transport (STILT) atmospheric transport modeling framework to validate Tier 1 findings without satellite or aerial data. Implementing this approach, we conducted comprehensive measurements across four major shale gas plays representing 84% of China’s total shale gas production in 2023. Our methodology integrates downwind stationary point measurements to quantify well pad emission rates and applies a bootstrapping technique to determine block-specific average emission levels. Following validation of the stationary monitoring results through STILT, extrapolation of findings from these four production plays enabled estimation of methane emissions from shale gas production in China. This research addresses a critical knowledge gap regarding China’s emission profile and provides essential scientific underpinning for developing targeted emission reduction policies.

## Methods

### Study Area

China’s shale gas industry is dominated by two state-owned giants: China National Petroleum Corporation and China Petroleum & Chemical Corporation, with their operations primarily concentrated in the Sichuan Basin. The terrain is predominantly hilly and mountainous, with burial depths ranging from 2,200 to 4,000 m.^25^ Our investigation covered four major production blocks that represent 84% of China’s total shale gas production: the Changning (5 bcm/year, 20%), Weiyuan (4.5 bcm/year, 18%), Fuling (8.5 bcm/year, 34%), and Luzhou (3 bcm/year, 12%) blocks, with Luzhou undergoing expansion targeting 10 bcm annual production.^26^ Field measurements were conducted in two phases: Fuling in August 2023 and the remaining blocks in July 2024. From an initial survey of 125 well pads (each containing 4–10 production wells), 90 pads met rigorous quality control standards: 27 in Changning, 22 in Weiyuan, 26 in Fuling, and 15 in Luzhou, accounting for 33%, 33%, 22%, and 32% of the well pads in the respective blocks. Collectively, the final data set covers about 21.5% of all active well pads in China. Based on proportional representation analysis, the data set effectively captures the overall well characteristics of the four shale gas blocks. Figure 1 illustrates the geographical distribution of the measurement sites across the Sichuan Basin.

**Figure 1:**
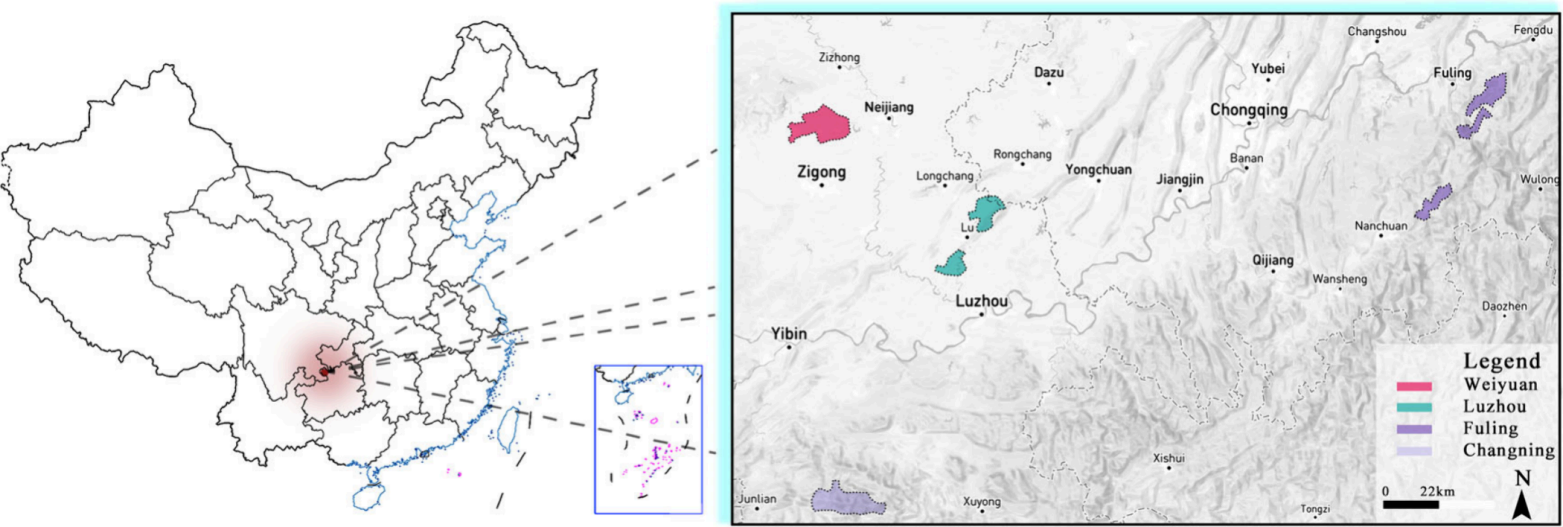
Geographical distribution of surveyed shale gas production blocks in the Sichuan Basin, China. The study area encompasses four major production blocks: Changning, Weiyuan, Fuling, and Luzhou.

### Sampling Strategy

Before the field campaign, we conducted several preliminary surveys to develop a comprehensive sampling strategy across all four shale gas blocks. The planned routes prioritized accessibility, targeting roads within a 100-m radius of well pad centers to maximize coverage. To ensure measurement accuracy, we used Google Earth imagery to identify potential sources of interference near the well pads. We excluded sites with nearby cattle or other interference sources. During the actual measurements, only two measurements were excluded due to interfering sources from nearby cattle: one in the Changning block and one in the Fuling block. Additionally, we bypassed locations lacking suitable downwind parking spots for measurement equipment. Furthermore, we would like to clarify that our selection of well pads was conducted randomly, and operators were not notified before the measurements. This approach minimizes the potential for operators to alter their operations during the measurement periods, thereby reducing the risk of selection bias and measurement error. This systematic approach to site selection and measurement planning helped maintain data quality while optimizing coverage across all blocks.

### Mobile Laboratory Platform

The research utilized a mobile laboratory platform equipped with multiple analytical instruments for comprehensive atmospheric measurements (Figure S1). A cavity resolved dissolution spectrometer (CRDS, model G2201-I, Picarro Inc.) operating in CO_2_–CH_4_ mode measured atmospheric concentrations of methane and CO_2_. It also provided data on the ratio of stable carbon isotopes of methane and CO_2_. Delays caused by air entering the CRDS through a section of PTFE tubing were considered during the subsequent data processing. Additionally, a CSAT3B 3D ultrasonic anemometer (Campbell Scientific Inc.) was installed on top of the mobile laboratory platform. This anemometer was used to collect information regarding wind direction and speed throughout the survey. Furthermore, a ZERO PLUS CH_4_ C_2_H_6_ H_2_O mid-infrared laser spectroscopy gas analyzer provided atmospheric methane and ethane concentration data to support source attribution. The platform also included a GN-168BT-GPS for real-time positioning and an R3–50 3D sonic anemometer for additional wind monitoring, enabling mobile measurements across the study area.

### Two-Tiered Mobile Measurement Approach

This study employed a two-tiered mobile measurement approach to quantify and validate methane emissions from China’s upstream shale gas production (Figure 2). Tier 1 utilized stationary downwind monitoring at well pads to quantify emission rates using the OTM-33A method. Tier 2 employed mobile measurements across production blocks to capture regionally integrated methane enhancement signals resulting from emission sources. Within the STILT atmospheric transport modeling, we constructed two methane emission fields based on Tier 1-derived emission rates : a Baseline scenario and a 10× Baseline scenario. Simulations were executed for both scenarios to generate methane concentration enhancements, which were then statistically compared against the enhancements observed from Tier 2 mobile measurements. This comparative framework was designed to evaluate whether downwind-derived estimates exhibit systematic underestimation, thereby yielding a more reliable quantification outcome than reliance on stand-alone downwind stationary monitoring.

**Figure 2:**
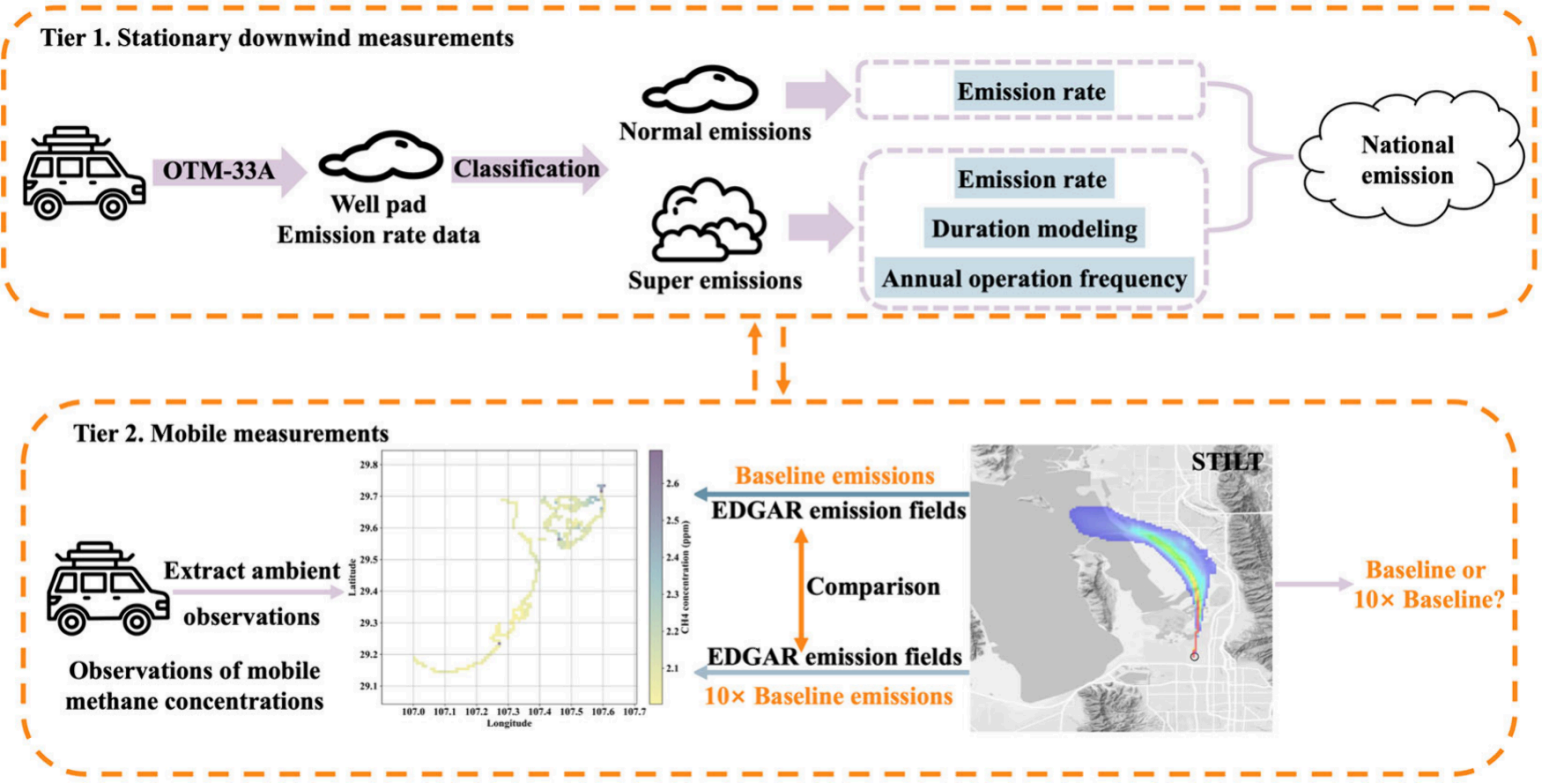
Schematic of the two-tiered mobile measurement methodology showing the integration of stationary downwind monitoring (Tier 1) and mobile measurements with STILT modeling validation (Tier 2).

#### Tier 1—Static Downwind Measurements

During the stationary downwind monitoring period, our vehicle-mounted mobile laboratory was positioned 30–100 m downwind of each target well pad for 15–20 min, with the engine turned off to prevent exhaust interference. The mobile laboratory also recorded ambient methane concentrations while moving slowly through the four blocks, providing validation data for the downwind measurement results.

We utilized the US EPA’s OTM-33A protocol , which employs the Gaussian plume model, to quantify methane emission rates. This approach allows for rapid assessment without site access, mitigated potential bias from operator-reported data and was implemented in five steps based on the standard point-source Gaussian dispersion assumptions.^19,27^

**1. Alignment:** the second-level data collected by the 3D acoustic anemometer were rotated to align with streamline coordinates for analysis. **2. Stability Classification:** the local stability class of wind conditions was determined during the static measurement period, based on two parameters: (i) the standard deviation of two-dimensional wind direction and (ii) turbulent intensity. Stability classes were obtained separately for each parameter, and their arithmetic mean determined the final stability classification, which provided essential parameters for subsequent calculations. **3. Diffusion Parameter Determination:** horizontal (σ_*y*_) and vertical (σ_*z*_) diffusion values were determined from point source dispersion tables based on the measured distances from the emission source and the local stability class of wind conditions (OTM-33A Appendix F1). These diffusion values quantify the atmospheric spreading of methane plumes. **4. Gaussian Modeling:** methane concentration *G*(*y*, *z*) was modeled using a Gaussian distribution in the measurement plane, as shown in eq 1.

1 $$G\left(y,z\right)=C_{peak}\cdot \exp\left(-\left(\frac{y^{2}}{2\sigma_{y}^{2}}+\frac{z^{2}}{2\sigma_{z}^{2}}\right)\right)$$

where *C*_*peak*_ is the peak methane concentration determined by Gaussian fitting of wind direction and methane concentration (g/m^3^); *y* and *z* are the horizontal and vertical distances (m), respectively; σ_*y*_ and σ_*z*_ are the horizontal and vertical standard deviations (m), respectively. The plane-integrated methane concentration (*G*, g/m) refers to the total concentration of methane, integrated over a specified plane. It was calculated as

2 $$G=C_{peak}\cdot \iint \exp\left(-\left(\frac{y^{2}}{2\sigma_{y}^{2}}+\frac{z^{2}}{2\sigma_{z}^{2}}\right)\right)=2\pi \cdot C_{peak}\cdot \sigma_{y}\cdot \sigma_{z}$$

The methane emission rate was obtained by multiplying the resulting plane integral methane concentration by the average wind speed, as indicated in eq 3:

3 $$Q=2\pi \cdot C_{peak}\cdot U_{a}\cdot \sigma_{y}\cdot \sigma_{z}$$

where *Q* represents the methane emission rate (g/s) and *U*_a_ is the average wind speed (m/s).

**5. Quality Control:** data screening was conducted on the methane emission rate data set based on measurement time, wind speed, turbulence intensity, and other relevant criteria. Data failing quality standards were flagged, and those exceeding the allowable number of flags were excluded from the data set (Supporting Information S2). The disqualification rates for the Changning, Weiyuan, Fuling, and Luzhou blocks were 25%, 27%, 21%, and 31%, respectively.

The calculation of methane emission rates using the OTM-33A method involves inherent uncertainties, which have been systematically evaluated through controlled methane release experiments across multiple studies. Halley et al.^18^ demonstrated that 72% of their measurements fell within ± 30% of the true emission rates, while Robertson et al.^28^ further quantified these uncertainties, reporting typical 1σ uncertainties of ± 28% in rate estimation. Based on the reproducibility of these uncertainty patterns across different experimental conditions and geographical settings, we adopted Robertson et al.’s^28^ uncertainty characterization model, which employs an asymmetric normal distribution to account for the systematic bias in the OTM-33A method, with standard deviations of +39% for positive errors and – 22% for negative errors. This asymmetric distribution better reflects the method’s tendency to underestimate emission rates and provides a more robust framework for uncertainty quantification in well pad emission measurements.

#### Tier 2—STILT Model Validation

Our study found that the leakage rate of shale gas production in China is an order of magnitude lower than that inmajor shale gas production regions like the U.S. While Tier-1 site-level quantification using the OTM-33A method offers valuable information, this approach is subject to substantial uncertainties at individual sites and potential systematic underestimation, as shown in previous studies.^27^ To independently validate our finding of a low leakage rate, we applied the atmospheric transport model STILT to analyze ambient methane measurements collected by a mobile platform traversing the shale gas region. Using these observations, we aimed to test whether the leakage rate instead aligned with the higher levels reported in previous studies in the U.S. (approximately an order of magnitude greater than our OTM-derived estimates). This analysis was designed to provide a semiquantitative assessment complementary to the Tier-1 analysis, rather than a precise flux quantification, because of the uncertainties in coarse-resolution modeling of local transport (see description below).

These mobile observations were measured when the vehicles traversed the shale gas blocks, hence capturing both source-impacted spikes and ambient baseline concentrations. While elevated methane spikes were impacted by nearby methane sources, the baseline concentrations represent the ambient methane concentrations of well-mixed air in the boundary layer. The latter contain the contribution from local emissions after mixing with the regional background air and therefore can be compared with STILT simulations to constrain the magnitude of local emissions.

To extract ambient observations from the raw mobile measurement data, the study area was gridded at a 0.01° × 0.01° resolution. The ambient methane concentration for each grid cell was defined as the 5th percentile of all measurements within it. Grid cells with fewer than 40 data points were excluded to ensure statistical robustness . The spatial minimum of these grid-cell concentration on each sampling day was determined as the regional background, which was subtracted to derive observed methane enhancements for comparison with STILT simulations.

The footprints (sensitivity of observed methane concentration to emissions in each grid cell) of ambient concentration measurements were simulated with a Lagrangian atmospheric transport model, STILT. The simulation was driven by the Global Forecast System (GFS) meteorological field. At the time and location of measurements, 1000 particles were released in the STILT simulation, and their transport was tracked backward in time for up to 1 day. The residence time of particles within the boundary layer of each grid cell was used for computing spatially–temporally varying footprints archived hourly at the 0.01° × 0.01° resolution in a 2° × 2° domain. Our pretests showed that 1 day back-tracking was sufficient for this analysis, because here we focused on the concentration gradient at scales of ∼ 1–10 km. Remote emissions transported for more than 1 day contribute minimally to these local gradients because they have already been sufficiently mixed at this spatial scale, and hence the local gradient is predominantly due to local emissions. Currently, our STILT simulations utilize coarse-resolution GFS meteorology data. While this approach effectively captures the influence of regional emissions on observed enhancements, it may introduce notable errors in local-scale transport. Future improvements could involve incorporating higher-resolution meteorological data to enhance accuracy in transport simulation and therefore the ability of the method to constrain methane fluxes.

To evaluate the consistency between the stationary monitoring results and the mobile observations, we performed STILT simulations under two methane emission scenarios: a baseline scenario and a 10× baseline scenario.The baseline emission field was constructed using the emission rates derived from the stationary downwind measurements, with unmeasured well pads randomly generated from a database based on measurement results. The 10× baseline scenario was derived by enhancing the baseline emission field by 10 times. To account for the influence of other sources, the emission field for the nonoil/gas sectors was obtained from the Emissions Database for Global Atmospheric Research (EDGAR). The simulated footprints were convolved with these emission fields to derive the methane enhancements, which were compared with observed enhancements as described above. We calculated the biases between the simulated and observed methane enhancements and established whether the baseline or 10× baseline scenario better agreed with the observations.

### Emission Estimation Methods

Based on field survey data, this study classified methane emissions from shale gas well pads in China into two distinct categories: (1) routine continuous emissions that persist throughout the well pad’s production cycle; (2) intermittent super-emissions, which are characterized by exceptionally high emission rates and contribute disproportionately to the total emissions, are often induced by gas lifting, incomplete combustion or venting of compressors, and downhole operations, etc. The thresholds for defining super-emitters were established as the top 4%, 5%, 10%, and 15% of emissions by contribution, respectively, to evaluate the influence of threshold selection on methane emission estimates. To account for the substantial differences in emission intensity and temporal patterns between these categories, we developed a composite model for total methane emissions:

4 $$E_{CH4}=\left(q_{n}\cdot t_{y}+q_{s}\cdot t_{s}\cdot r\cdot \lambda_{s}\right)\cdot N_{pad}/1000$$

where E_CH4_ represents total methane emissions (ton) from shale gas production in China in 2023; q_n_ denotes the routine emission rate (kg/h); t_y_ is the annual duration, 8,760 h; q_s_, t_s_, r, and λ_s_ characterize supere-missions through their rate (kg/h), super-emitter duration (h), temporal proportion, and annual super-emitter frequency, respectively; N_pad_ is the number of well pads in Sichuan Basin.

For emission rate estimation (q_n_ and q_s_), we employed bootstrapping analysis, which is a robust statistical technique particularly suitable for small-sample data sets. It provided valuable statistical information such as distribution shape, peak, and mean, without making assumptions about specific distributions.^19,28^ The method involves generating multiple replicate data sets through random sampling with replacement from the original observations. This resampling process is repeated thousands or even millions of times. From these resampled samples, statistical parameters such as the mean can be calculated to estimate the parameter distribution and confidence interval. In our analysis, the q_n_ per well pad across the four production blocks were derived from block-specific survey data sets. Conversely, the q_s_ was calculated from a pooled data set integrating all four production blocks, as only 16 operation-induced super-emission events were documented.

In the bootstrapping analysis, we first established a normal distribution for the original data set, with the mean representing the actual measured values and a standard deviation derived from +39%/–22% of these values to simulate the measurement error. We then performed resampling with replacement on the original data set to generate 100,000 simulated data sets, maintaining the same sample sizes as the original data set (Changning: 22, Weiyuan: 20, Fuling: 24, Luzhou: 9, super-emissions: 16). After calculating the mean for each simulated data set, we aggregated these means to form an empirical distribution, allowing us to determine the mean emission rate and the 95% confidence interval for the watershed well pads.

The Monte Carlo simulation is employed to quantify annual methane emissions from super-emissions. The model integrates three core modules: (1) Emission rate modeling. The bootstrapping methodology has been introduced in preceding sections; (2) Duration modeling. The super-emitter duration (t_s_) and super-emission temporal proportion (r) are defined as triangular distributions: Triangular (min = 8, mode = 64, max = 120) and Triangular (0.4, 0.6, 0.8), respectively.^29^ (3) Annual super-emitter frequency modeling. The modeling of annual super-emitter frequency­(λ_s_) per well pad was constructed based on field survey data (e.g., 27 well pads investigated among 82 in Changning Block, identifying 3 super-emitter). The Wilson score interval method was employed to establish the 95% CI for super-emitter frequency p. This probability estimate was integrated with engineering constraints, λ_s_×t_s_×r = p × 365. Finally, 100,000 Monte Carlo simulations were executed to quantify the annual super-emission flux and its 95% confidence interval.

### Methane Leakage Rate

The methane leakage rate (Q_leak_) is defined as the percentage of methane emitted into the environment relative to the methane produced:

5 $$Q_{leak}=E_{CH4}/P_{CH4}$$

where P_CH4_ refers to the annual methane production (t), calculated as follows:

6 $$P_{CH4}=P_{methane}\cdot X\cdot 44.64\;\left(mol/m^{3}\right)\cdot 0.000016\;\left(t/mol\right)$$

where P_methane_ represents annual production of shale gas (m^3^), and *X* denotes the percentage of methane in shale gas, 97.81%. Given that *X* misestimation can bias Q_leak_ calculations,^30^ we have employed empirical measurements from established literature for this parameter in our computations.^31–34^

## Results and Discussion

### Ethane:Methane Ratios

We analyzed the ethane-to-methane (C2:C1) ratio, a widely utilized tool in the oil and gas industry for fingerprinting shale gas characteristics, across four production blocks: Changning, Weiyuan, Fuling, and Luzhou. Selected methane results exceeding the background concentration and corresponding ethane concentrations were plotted (Figure S3) and subjected to fitting analysis. The results revealed distinct C2:C1 ratios for each block: Changning at 0.398 (0.393–0.404)%, Weiyuan at 0.541 (0.536–0.546)%, Fuling at 0.586 (0.584–0.588)%, and Luzhou at 0.298 (0.292–0.304)%. These values are significantly lower than those typically observed in U.S. shale gas production blocks (Figure 3a),^35–37^ reflecting fundamental differences in gas composition between the regions. The lower ratios in Chinese shale gas production can be attributed to the unique characteristics of Chinese shale gas deposits. Unlike U.S. formations, Chinese shale gas exists primarily in dry gas form, with no associated shale oil production. The gas composition typically consists of 96.4%–98.6% methane and 0.32%–0.70% ethane, resulting in very low C2:C1 ratios in captured emissions.^32^ In contrast, U.S. shale gas typically contains higher ethane proportions, with dry gas containing over 1% ethane. In areas with concurrent shale oil production, the ethane proportion in wet gas can exceed 10%.^36–38^ These compositional differences underscore the distinct geological and production characteristics of Chinese and U.S. shale gas resources.

**Figure 3:**
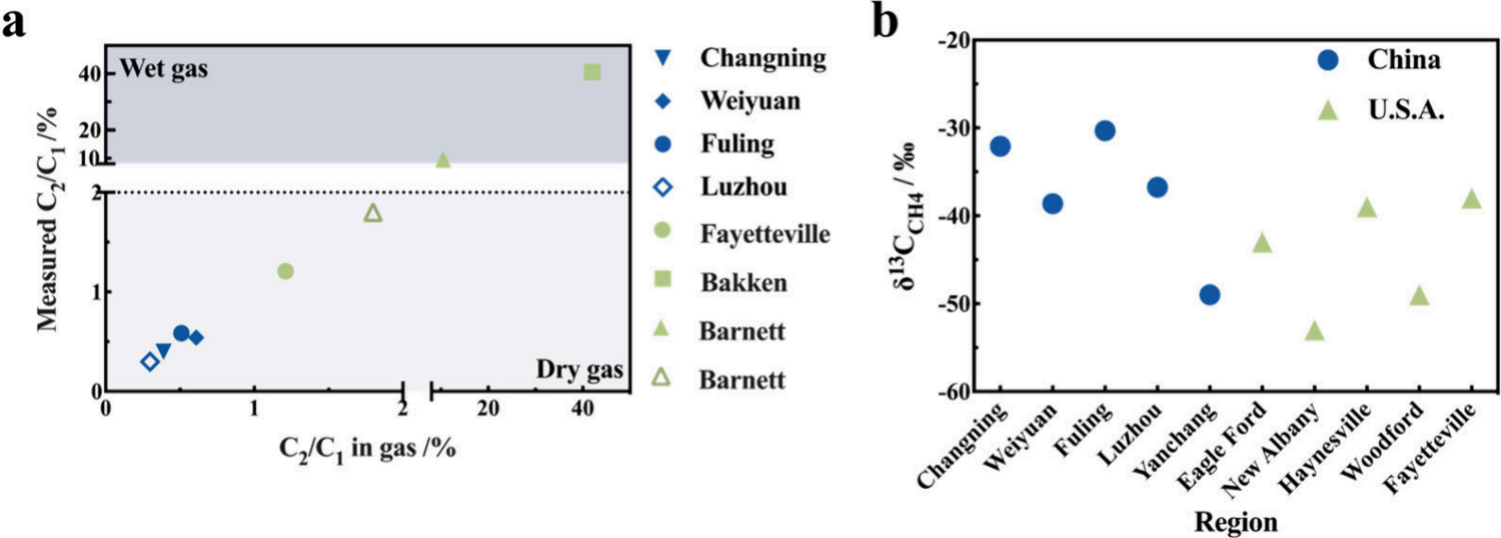
Comparison of C2:C1 ratio and isotopic signatures across shale gas production blocks. (a) Ethane-to-methane (C2:C1) ratios showing distinct differences between Chinese and U.S. production regions. (b) Keeling plots demonstrating enriched δ^13^C_CH4_ values in Chinese shale gas blocks.

### Isotopic Signatures

In addition to the C2:C1 ratio, this study also employed δ^13^C_CH4_ isotopic signatures as a complementary tool for determining methane sources in the atmosphere, given that different sources exhibit distinct carbon isotopic characteristics. Research by Schwietzke et al. indicates that the global mean δ^13^C_CH4_ values for two major source categories: approximately – 44.0‰ (fossil fuel) and – 62.2‰ (biogenic).^39^ However, there are notable variations in δ^13^C_CH4_ among different fossil fuel-producing regions; for example, the δ^13^C_CH4_ in the New Albany of the United States is approximately – 53‰, while in the Eagle Ford, it is approximately – 43‰.^38^

Our analysis of four Chinese shale gas production blocks revealed notably enriched δ^13^C_CH4_ values: Changning at – 32.10‰ (−33.18‰ to – 31.01‰), Weiyuan at – 38.62‰ (−39.47‰ to – 37.77‰), Fuling at – 30.33‰ (−30.81‰ to – 29.84‰), and Luzhou at – 36.75‰ (−39.05‰ to – 34.46‰), as detailed in Figure S4. These measurements, obtained using the Keeling plot approach (see Supporting Information, SI), showed significantly higher enrichment compared with U.S. shale gas regions (Figure 3b). The distinctive isotopic enrichment observed in Chinese shale gas can be attributed to isotopic reversal, where the typical pattern of δ^13^C_1_ < δ^13^C_2_ < δ^13^C_3_ shifts to δ^13^C_1_ > δ^13^C_2_ > δ^13^C_3_.^38^ This phenomenon is typically observed in mature gases, where the δ^13^C of C_1_ exceeds approximately – 43‰, and the dry gas ratio C_1_/∑(C_1_–C_5_) is usually greater than 95%. Several mechanisms have been proposed to explain the cause of isotopic reversal, including variations in gas sources,^40^ Rayleigh-type fractionation,^41,42^ and water–kerogen redox reactions.^41^

### Two-Tiered Quantification of Methane Emission Rates

After conducting a statistical analysis of the emission data from the four shale gas production blocks, the cumulative emission distribution was plotted (Figure 4). The emission rates demonstrated a pronounced skewed distribution pattern, aligning with findings from previous research in this field. Subsequently, a log-normal distribution was fitted to the emission rate data from the well pads. Specifically, the Anderson–Darling (AD) test for Changning yielded a statistic of 0.31 and a corresponding P-value of 0.54. This suggests that, at the significance level of α = 0.05, we cannot reject the hypothesis that the data follow a log-normal distribution. Similar log-normal distribution patterns were observed in the emission rates from Weiyuan, Fuling, Luzhou and super-emissions.

**Figure 4:**
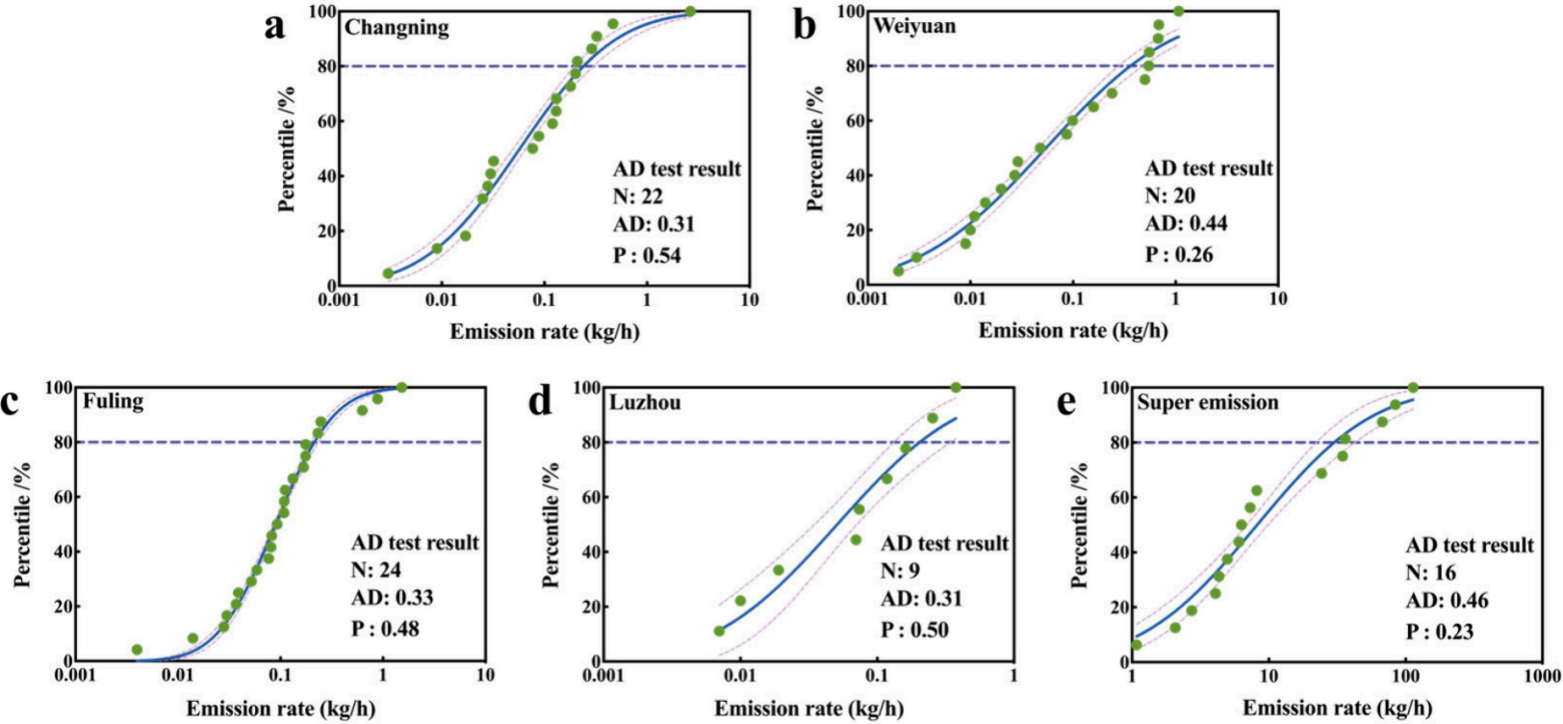
Cumulative probability plots of methane emission rate distributions and Anderson–Darling (AD) test results for Changning (a), Weiyuan (b), Fuling (c), Luzhou (d), and Super-emissions (e).

To verify that our findings from the Tier 1 did not severely underestimate emission rate, we employed the STILT atmospheric transport model. We compared observed methane enhancements with two emission scenarios: (1) methane emission rates derived from downwind static measurements (baseline) and (2) an alternative high-emission scenario assuming emission rates 10 times greater (10× baseline). While STILT simulations carry inherent uncertainties, our observational system combining STILT and mobile observations could effectively distinguish the differences of concentration enhancement due to different emission rates based on our simulation (Figure 5).

**Figure 5:**
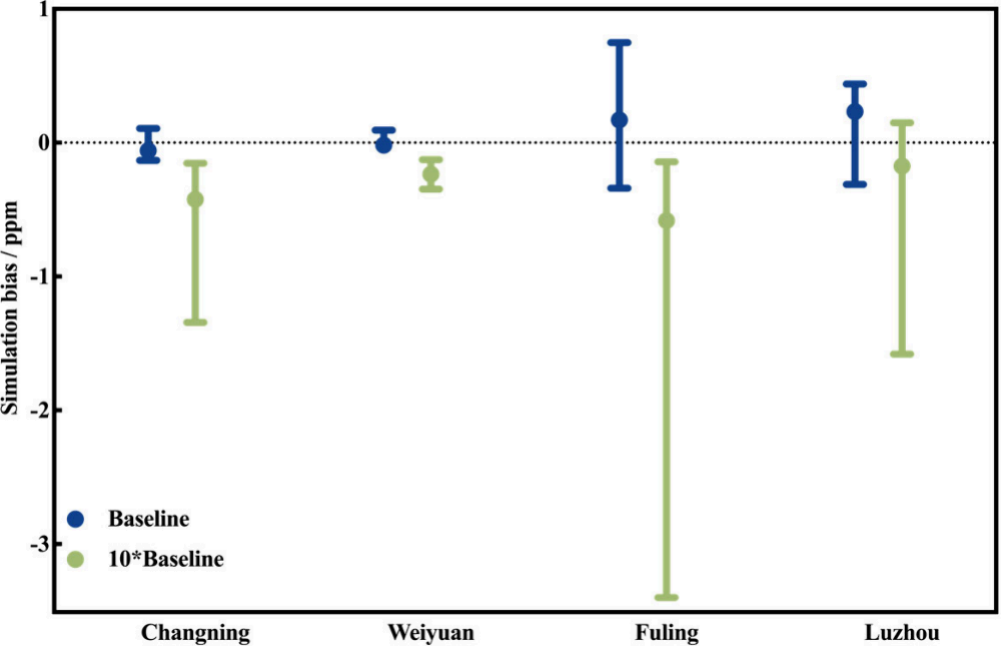
Key parameter statistics of the observation–simulation bias distribution for methane-emission-sensitive grid cells under the two emission scenarios are presented, where the points, lower limit, and upper limit represent the median, 5th percentile, and 95th percentile, respectively.

Analysis of observation–simulation biases at the 0.01° × 0.01° grid demonstrates statistically superior consistency between the observed methane enhancements and the baseline scenario across all four shale gas blocks. Specifically, the 90% confidence intervals of the observation–baseline simulation bias encompassed 0 in all blocks, confirming nonsignificant systematic deviations under the baseline scenario. However, in Changning, Weiyuan, and Fuling, the observation–10× baseline simulation bias intervals were consistently negative, suggesting overestimation of methane enhancements by the 10× scenario. While Luzhou showed 0-encompassing biases for both scenarios, the 10× baseline spread was significantly larger, suggesting that the emission rate in Luzhou may have been underestimated slightly, and the emission rates are more likely to fall between 1× and 10×. This phenomenon can be attributed to the high-intensity emission events triggered by more frequent operations in Luzhou. Due to their high release altitude, such emission sources often cannot be detected by conventional ground-based monitoring. Please refer to the SI for additional analyses regarding the spatial distribution of observation–simulation biases, detailed bias distributions across the various blocks, and sensitivity tests for STILT modeling configurations.

Bootstrapping-derived emission rates were quantified as follows: Changning at 0.23 (0.12–0.37) kg/h, Weiyuan at 0.24 (0.12–0.38) kg/h, Fuling at 0.21 (0.10–0.36) kg/h, and Luzhou at 0.12 (0.06–0.19) kg/h (inclusive of all subdetection-limit well pads), with super-emissions exhibiting a characteristic emission rate of 25.42 (10.63–43.76) kg/h. The analysis of emission patterns revealed a stark contrast between numerous small emission sources and a few dominant super-emitters in the shale gas production blocks. While small sources had a limited overall impact, the top 10% of emitters (with emission rates ranging from 6.0 to 98.9 kg/h) accounted for 89% of total emissions across all four blocks, with individual block contributions ranging from 62% to 94%. Significant regional variations were observed in emission distributions, with Changning, Weiyuan, and Fuling showing 80% of emission rates below 1 kg/h, while Luzhou demonstrated notably higher rates with 80% below 10 kg/h. This elevated emission profile in Luzhou can be attributed to its status as the most recently developed block, currently under construction with numerous ongoing engineering activities; the associated facilities are not yet fully established, resulting in direct emissions following gas lift operations. Additionally, the operation of numerous compressors has contributed to substantial methane emissions. These activities have been extensively documented through video footage, with detailed documentation available in the SI.

### Methane Emissions from China’s Shale Gas Production

This study employs Tier 1 activity’data from China’s four major shale gas production blocks, validated through Tier 2 verification methods, to independently model routine emissions and operation-induced super-emissions. Subsequently, this information was combined with the number of well pads in each block to assess the national methane emissions from shale gas production. The results are presented in Table 1. The total methane emission by China’s shale gas production sector was estimated at 14,254–17,393 t. With China’s 2023 shale gas production at 250 bcm and a methane content of 97.81%, the Q_leak_ was 0.08%–0.10%. This study observed that the impact of super-emitter threshold selection on the overall emission estimate is mitigated by an inherent compensatory mechanism. Specifically, raising the threshold (e.g., to 30 kg/h) leads to a marked reduction in the frequency of events classified as super-emitters, while the emission intensity of sources thereby reclassified into the routine category increases correspondingly. These opposing trends offset each other, resulting in a final aggregate emission estimate that remains largely insensitive to variations in the threshold definition. Notably, the Q_leak_ is significantly lower than those observed in U.S. shale gas production blocks, particularly those in wet gas regions.^13,43,44^

**Table 1:** Summary of Mean Emission Rates, Annual Emissions, Q_leak_, and Other Relevant Information with 95% CI in 2023

Threshold setting	Mean emission rate q_n_, kg/h	Mean emission rate q_s_, kg/h	Annual emissions, t/y	Q_leak_, %
Top 4%, 30 kg/h	1.01 (0.48–1.76)	59.3 (45.8–73.7)	17,393 (5,922–31,435)	0.10 (0.03–0.18)
Top 5%, 20 kg/h	0.74 (0.42–1.14)	52.3 (38.8–67.2)	16,842 (6,444–29,991)	0.10 (0.04–0.17)
Top 10%, 6 kg/h	0.44 (0.25–0.67)	32.1 (18.3–48.6)	15,278 (6,379–28,297)	0.09 (0.04–0.16)
Top 15%, 2 kg/h	0.19 (0.13–0.26)	20.5 (8.6–35.7)	14,254 (5,316–27,853)	0.08 (0.03–0.16)

### Possible Reasons for Lower Methane Leakage Rate

Understanding methane leakage rate is crucial for assessing industry management and optimizing production processes. To further explore the reasons for the relatively low Q_leak_ in China’s shale gas production bases, we compared them with several ground monitoring studies from U.S. shale gas regions.^18,19,28,44^ The comparison revealed that China’s Q_leak_ remains notably lower, approximately 1 order of magnitude below U.S. levels, as shown in Figure 6.

**Figure 6:**
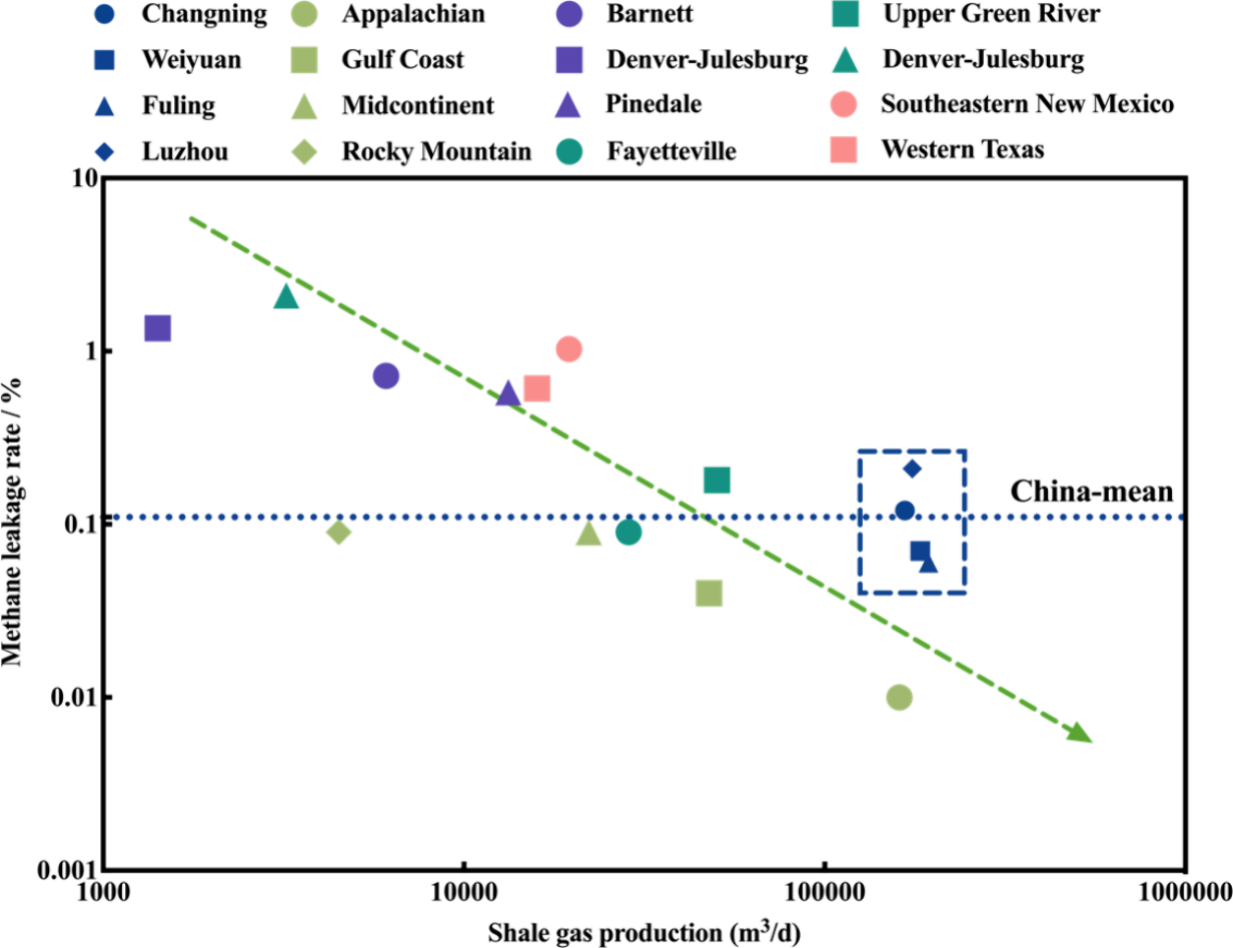
Q_leak_ in different shale gas production regions. This study compared the Q_leak_ from shale gas wells in four blocks with several ground-based monitoring studies. The blue, light green, purple, dark green, and red data points represent results from this study, Allen et al.,^44^ Brantley et al.,^18^ and two studies by Robertson et al.,^19,28^ respectively.

To elucidate the intrinsic mechanisms behind the relatively low Q_leak_ in Chinese shale gas production, we systematically examined the disparities in shale gas production systems between China and the United States. The Q_leak_ calculation model indicates that both shale gas production and methane emission rates jointly determine the ultimate methane leakage levels. Existing research demonstrates a significant positive association between methane emission rates and shale gas production; nevertheless, this relationship is not linear. Instead, it is predominantly influenced by super-emission events resulting from facility malfunctions or human operations. Notably, significant variations exist in production capacities across different shale gas blocks. As illustrated in Figure 6, a systematic compilation of Q_leak_ and production data from key shale gas regions in China and the U.S. reveals that the maximum-to-minimum production ratio of individual well pads can exceed 130-fold. This phenomenon underscores the critical role of well pad production levels in influencing Q_leak_, with higher production levels often associated with lower Q_leak_.

Chinese shale gas production is characterized by several notable features. First, it represents a typical dry gas production system, with average production rates per well pad significantly surpassing those in major U.S. regions, particularly those in U.S. areas dominated by wet gas production. Second, the absence of the need for additional separation and storage equipment fundamentally reduces potential leakage risks.^28^ Furthermore, dry gas production avoids the flash emissions associated with wet gas separation processes and decrease frequent liquid unloading operations.^19^ The synergistic effects of these factors contribute to the maintenance of low Q_leak_ in Chinese shale gas production. It is important to emphasize that these differences primarily stem from the inherent distinctions in resource endowments between the two countries, rather than differences in management practices. For instance, while the Q_leak_ in U.S. wet gas regions is generally higher than that in Chinese shale gas regions, the Q_leak_ in the Fayetteville dry gas region of the U.S. is 0.09%, comparable to China’s overall Q_leak_. This suggests that measuring Q_leak_ using energy-normalized metrics offers broader applicability and comparability than traditional volumetric or mass-based standards. This finding contributes to standardizing the evaluation of methane releases from the global oil and gas production.

This study is constrained by the limited sample size of intermittent super-emission data, which precluded a classified analysis of emissions from different sources. The OTM-33A method employed in this study exhibits inherent technical limitations, including relatively low sensitivity to high-intensity emission sources. As a single-pass measurement technique, it may not adequately capture intermittent, short-duration, high-intensity super-emission events.^19,45,46^ These constraints likely result in systematic underestimation of total emissions. Future methane emission assessments would benefit substantially from enhanced observation and modeling of super-emitters.^47^ This requires high-frequency, close-range measurements across diverse process conditions, equipment types, and operational states, particularly for known super-emission sources such as gas lift venting, compressor venting, and incomplete combustion. Systematic data collection would enable refinement of critical model parameters including event duration distributions, emission rate ranges, and occurrence frequencies. We recommend integrating complementary monitoring approaches, including aerial remote sensing, satellite-based inversion, and continuous ground-based sensors, to achieve comprehensive spatial and temporal coverage. When combined with facility operational logs and equipment-specific emission factors, these multiscale observations could yield more robust emission inventories. Although our current methodology provides a valuable baseline for China’s shale gas emissions, implementing these enhancements would substantially improve both the accuracy of emission quantification and the reliability of regional extrapolations.

### Super-Emitter Analysis and Characterization

Our investigation indicates a limited number of super-emitters account for 79.6% of total methane emissions. This finding corroborates previous research conclusions, further highlighting the importance of super-emitters in the quantification of methane emissions.^48^ These super-emitters typically exhibit intermittent emission characteristics, making them difficult to capture effectively in snapshot surveys and leading to overall emission underestimation.

Through detailed site investigations and photographic documentation, we identified that supere-missions primarily originate from gas lifting, incomplete combustion or venting of compressors, and downhole operations. In our survey, incomplete combustion from mobile gas-driven compressors and gas lift venting accounted for 23.2% and 33.0% of total emissions, respectively. Our observations at three well pads (two in Changning and one in Luzhou) revealed significant methane emissions due to incomplete combustion, with rates of 4.31, 34.63, and 36.25 kg/h, respectively. Additionally, in the Weiyuan block, we recorded a methane emission rate of 6.30 kg/h from a compressor during operations. Although we could not access this well pad for further investigation, we conservatively speculate that the emissions likely originated from stationary compressor exhaust or incomplete combustion processes. We observed two instances of direct emissions from gas lifting, with emission rates of 8.17 kg/h and 98.86 kg/h (Mean value after two repeated measurements: 84.08 and 113.64 kg/h) in the Luzhou block. These high emission rates may be attributable to the presence of numerous well pads in the early construction phase within the Luzhou block, where infrastructure such as three-phase separators and gas recovery systems is still inadequate and regulatory oversight is lacking. Due to the lack of production information from the well pads and our inability to access them for in-depth investigations, some super-emissions caused by activities such as workovers are difficult to fully document. For instance, in the Changning block, we observed one well pad with an emission rate of 6.05 kg/h during workover operations, during which multiple wells experienced significant production decreases. The producer is currently investigating the cause by deploying cameras in the well. These observations underscore the complexity of capturing and quantifying super-emitter events in varied operational conditions. The emission snapshots from these well pads are available in the SI.

### Policy Implications

This pioneering comprehensive study of methane emissions from China’s shale gas production, utilizing a two-tiered mobile measurement approach, provides crucial insights for guiding sustainable industry development and policy formulation. Our findings reveal a highly skewed emission distribution, with 61% of sources emitting below 0.20 kg/h, while the top 10% of sources account for 89% of total emissions. This distribution pattern emphasizes the critical importance of identifying and addressing super-emitters for effective emission reduction in the upstream shale gas industry.

The comprehensive reduction of methane emissions in China’s shale gas industry relies on a multidimensional collaborative governance system involving the government, operators, and third-party organizations.^49,50^ The core of this system lies in the organic integration of systematic policy design, leak detection and repair (LDAR), and a transparent supervision system, aiming to overcome the institutional and technical bottlenecks currently present in emission reduction practices. The government needs to strengthen top-level design and institutional constraints, establishing phased reduction targets based on the “Methane Emission Control Action Plan” and creating a dual-track regulatory model with a *baseline reward and punishment* mechanism. Specifically, methane can be incorporated into quota management through a carbon emission trading system, while projects that adopt low-carbon technologies such as electric-driven compressors and electric actuators should be incentivized. Operators should establish a multilevel methane leakage prevention and control system based on *continuous monitoring–periodic verification* to efficiently complete LDAR tasks. Although continuous monitoring systems may systematically underestimate emissions, prioritizing their deployment at production sites can improve the reporting of super-emission events, preventing them from escalating.^51^ Periodic verification should be designed with differentiation based on facility characteristics and operational stages, focusing on well pads that include compressors and those in construction stages.^52^ Independent verification by third-party organizations is a key aspect of enhancing governance effectiveness. By constructing an integrated monitoring network that combines satellite, aerial, and ground data, we can overcome the limitations of single data sources and provide transparent results for assessing methane emissions from shale gas production.^53,54^

Future research should evaluate the effectiveness of current emission reduction measures, identify areas for improvement, and monitor new initiative outcomes. Success in achieving sustainable development goals in China’s shale gas industry will require ongoing collaboration between industry stakeholders, academic researchers, and regulatory bodies, combined with robust policy implementation. This coordinated approach is essential for aligning the industry with sustainable development principles and contributing to global climate change mitigation efforts.

## Supplementary Material



## Notes

Detailed production information on the well pads as well as additional methods, including bootstrapping method, and a supplementary introduction to the two-tiered mobile measurement approach (PDF)
