# Supplementary material for: Assessing Methane Emissions from Shale Gas Production in China: A Two-Tiered Mobile Measurement Approach
Source: Environ Sci Technol. 2025 Dec 23;60(1):364–75. doi: 10.1021/acs.est.5c01953 (PMC12810235; doi:10.1021/acs.est.5c01953)
Supplement: Supplementary file 1 [file es5c01953_si_001.pdf]

Supplemental Information

Assessing Methane Emissions from Shale Gas Production in China: A Two-tiered Mobile Measurement Approach

Pu Hong, Yuzhong Zhang, Wenrui Shi, Shuang Zhao, Xin Feng,
Minghao Zhuang, Xi Lu and Meiyu Guo,

TABLE OF CONTENTS

S1. Mobile laboratory platform 2

S2. Data Screening 3

S3. Sensitivity Analysis of the STILT Model 4

S4. Fitting of the C_2:C_1 Ratio 5

S5. Keeling Plot Approach 6

S6. Validation of emission rate and methane loss rate 7

S7. Emission rate data 10

Supplemental Reference 12

Contents (12 pages):

Tables: S1-S3

Figures: S1-S6

Supplemental references

S1. Mobile laboratory platform

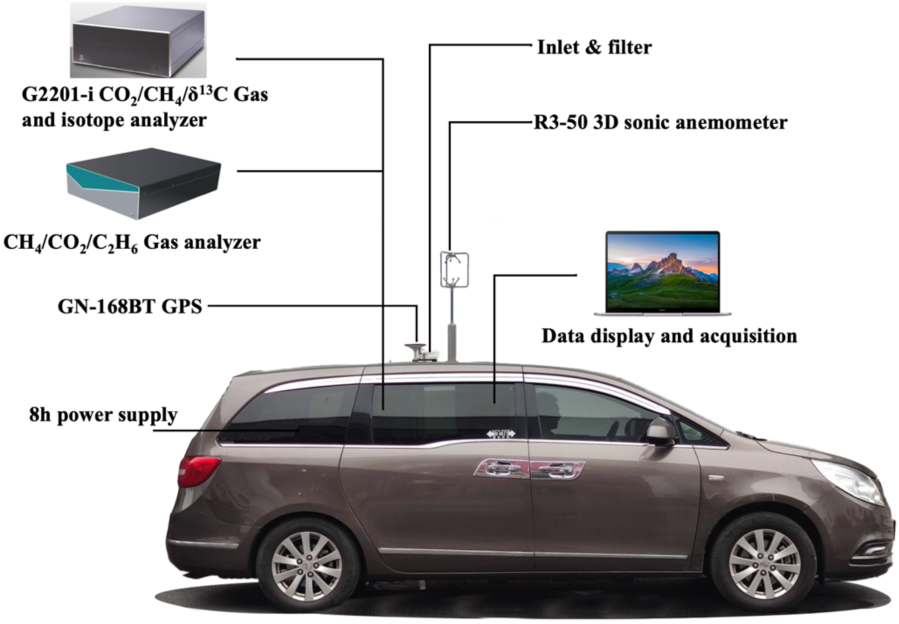

Figure S1. Mobile laboratory platform.

S2. Data Screening

When using the OTM-33A method, the methane emission rate survey results from the four blocks are categorized into three classes based on the data quality indicators (DQI) in Table S1. Class 1 measurements have fewer than 5 flags, Class 2 measurements have fewer than 10 flags, and Class 3 measurements have 10 flags or more. In the absence of multiple emission sources or wind field errors, data in Classes 1 and 2 are considered valid. Class 3 data are deemed invalid until further analysis is conducted. If low methane is the main reason for the data quality flags and the total number of flags is below 15, the data will be validated and included; otherwise, the data will be excluded.

Table S1. Data quality indicators for data Screening

DQI name	List of exceedance levels	Flag point value
Count	< 15 minutes of measurement	3
Wind direction	$(\theta_y+\theta_{\text{vertical wind}})/2 > 30$	1
	$2(\theta_y-\theta_{vertical\ wind})/(\theta_y+\theta_{vertical\ wind}) > 0.5$	1
Turbulent intensity	TI > 0.22	1
Average wind	$\bar{U} < 1.5$ m/s	1
speed	$\bar{U} < 1$ m/s	5
Wind variance	Wind variance > 2.5	5
	Wind variance > 5	10
Binned data	Highest concentration bin ≠ 180 ± 30°	1
	Highest concentration bin ≠ 180 ± 60°	3
Gaussian fit	R < 0.95	5
	R < 0.90	10
Methane level	Background methane < 1.7 ppm	1
	CH_4 enhancement < 0.15 ppm & Dist. < 50 m	10
	CH_4 enhancement < 0.15 ppm & Dist. 50–100 m	5
	CH_4 enhancement < 0.15 ppm & Dist. 100–150 m	3
	CH_4 enhancement < 0.15 ppm & Dist. > 150 m	1
	CH_4 enhancement < 0.1 ppm & Dist. < 50 m	10
	CH_4 enhancement < 0.1 ppm & Dist. 50–100 m	5
	CH_4 enhancement < 0.1 ppm & Dist. 100–150 m	3
	CH_4 enhancement < 0.1 ppm & Dist. > 150 m	1

S3. Sensitivity Analysis of the STILT Model

We conducted two sensitivity analyses: (1) a 12-hour backward tracking and (2) independent meteorological data. The results of these sensitivity tests, illustrated in Fig. S2, demonstrate a clear distinction between the 1× and 10× emissions under these perturbations, thereby reinforcing our findings. The independent meteorological data were derived from a WRF simulation that was nudged to GDAS data, employing a 12 km grid resolution over China.

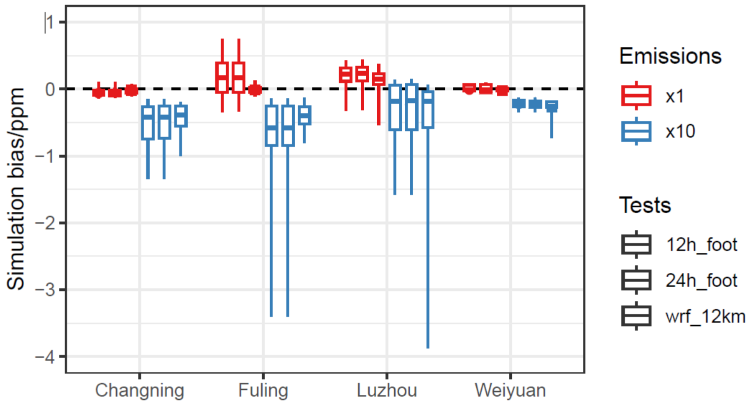

Figure S2. The sensitivity tests for STILT-based analyses using GFS data and 12 hour backtracking (12h_foot), GFS data and 24 hour backtracking (24h_foot; main results), WRF data and 24 hour backtracking (WRF_12km). Statistics of the observation-simulation bias distribution for methane emission-sensitive grid cells under the two emission scenarios are shown. The median line, lower quartile, upper quartile, lower whisker, and upper whisker represent the 50%, 25%, 75%, 5%, and 95% percentiles of the data, respectively. The WRF simulations were performed in 12 km grid and nudged to GDAS meteorological data.

S4. Fitting of the C_2:C_1 Ratio

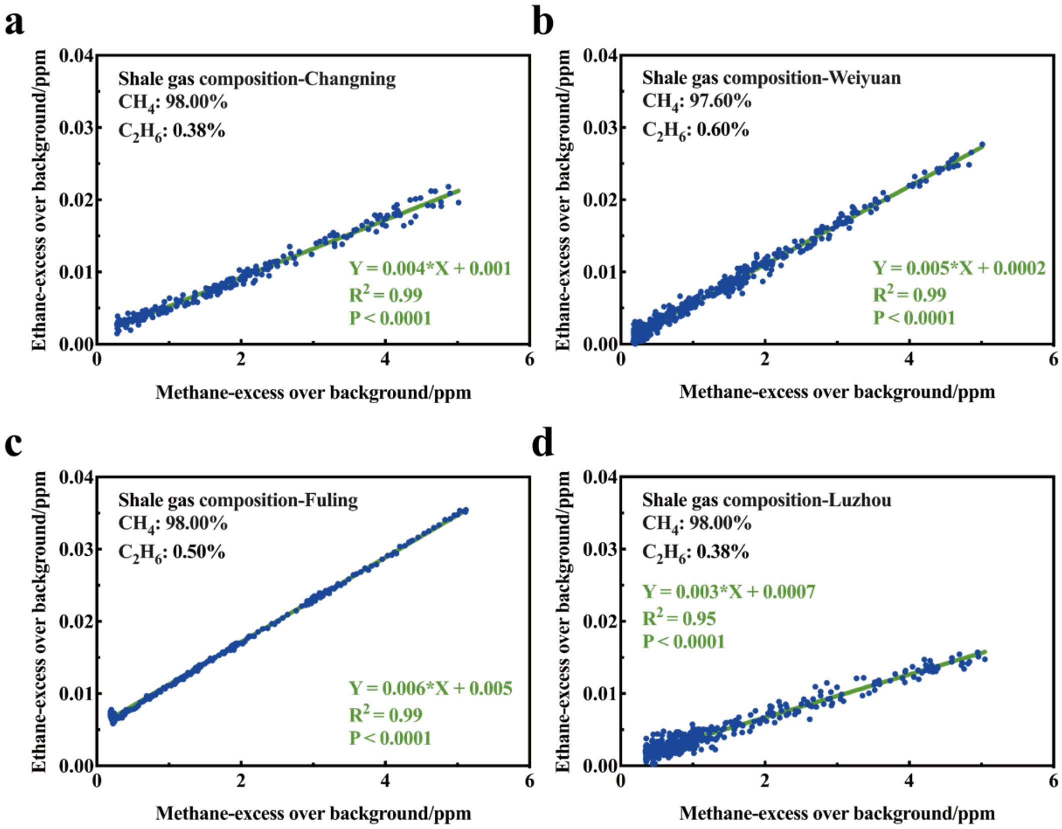

Figure S3. Fitting of the C_2:C_1 Ratio in China's Shale Gas Production Areas. The background concentrations of methane and ethane are defined as the 5th percentile of methane ambient concentrations measured during the survey. Pictures a–d correspond to the Changning, Weiyuan, Fuling, and Luzhou blocks, respectively. The slope of the fitting expression represents the C_2:C_1 ratio for each respective block.

S5. Keeling Plot Approach

The Keeling Plot Approach is used to determine the source $\delta^{13}C_{CH4}$ signature of methane emissions from shale gas fields. It involves plotting the inverse of the atmospheric methane concentration ($1/c_a$) on the X-axis and carbon isotopic ratios of the atmospheric methane($\delta^{13}C_a$) on the Y-axis. By analyzing the intercept of the plot, the $\delta^{13}C$ of the methane source can be determined[1,2]. In the context of the Fuling shale gas field, the study assumes that the main release source of methane is from the shale gas field itself. The concentration of atmospheric methane (c_a) is the sum of the background methane concentration (c_b) and the additional concentration component produced by the source (c_s), as shown in Eq (S1).

$$c_a = c_b + c_S \tag{S1}$$

Based on the principle of conservation of mass, we can get Eq. (S2):

$$\delta^{13}C_a c_a = \delta^{13}C_b c_b + \delta^{13}C_S c_S \tag{S2}$$

where $\delta^{13}C$ represents the carbon isotope ratio of each methane component. Combining Eq (S1) and (S2), $\delta^{13}C_a$ can be calculated using Eq (S3). By plotting $1/c_a$ and $\delta^{13}C_a$, the intercept obtained on the plot represents the $\delta^{13}C_{CH4}$.

$$\delta^{13}C_a = c_b \cdot (\delta^{13}C_b - \delta^{13}C_S) \cdot (1/c_a) + \delta^{13}C_S \tag{S3}$$

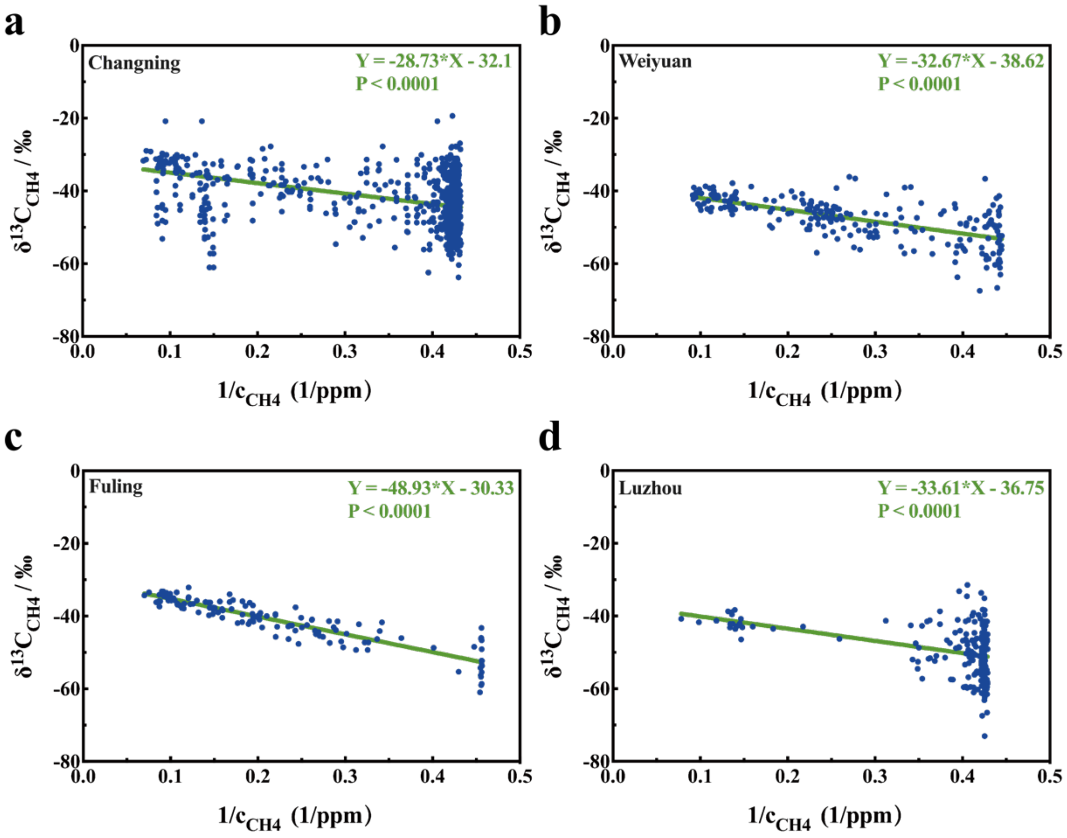

Figure S4. Keeling plot. Pictures a–d correspond to the Keeling plots for Changning, Weiyuan, Fuling, and Luzhou, respectively. The intercepts of the fitted lines represent the $\delta^{13}C_{CH4}$ for each respective block.

S6. Validation of emission rate and methane loss rate

We take the Fuling Block as an example to illustrate the data processing procedure. To extract ambient observations from raw mobile measurements, we find low-percentile methane measurements within a spatial and temporal window. First, we discretize the study domain on a 0.01°×0.01° grid and group together continuous mobile measurements by grid cell. We then compute the 5th percentile of data in each group to determine the ambient methane concentrations that are representative of the 0.01°×0.01° grid cells. The grid cells with the number of measurements less than 40 are discarded in this analysis. Fig. S5b illustrates the extraction of ambient signals from the raw mobile data. The extracted ambient data (red line) do not contain spikes impacted by nearby sources, while keeping the information of concentration gradients on a spatial scale corresponding to the 0.01°×0.01° grid (Fig. S5a).

To subsequently compare the consistency between the environmental methane concentration distributions under the two emission scenarios and the observation results, we determined the grid cells that are sensitive to shale gas emissions. As shown in Fig. S5c, the methane enhancement under the 10×baseline scenario was simulated, and statistical analysis showed that the number of grid cells sensitive to methane emissions was 52. Additionally, we simulated the environmental methane enhancement caused by the non-oil/gas sectors in Fig. S5d.

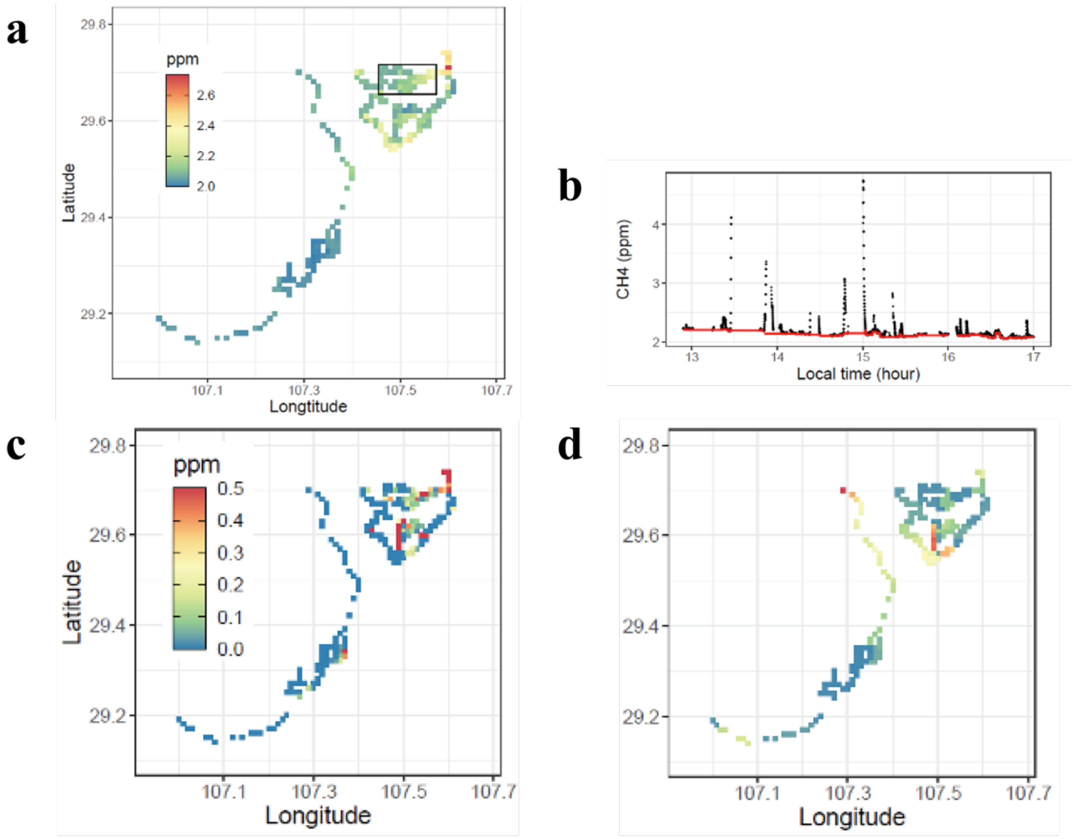

Figure S5. Extraction of ambient observations and identification of grid cells sensitive to shale gas emissions. (a) Spatial distribution of ambient methane concentrations derived from

continuous mobile measurements over the shale gas production region. (b) Illustration of the procedure separating ambient methane measurements from source-impacted measurements. Raw mobile measurement data are shown in black dots and derived ambient data are in red lines. Rectangle in the Fig. S5a corresponds to the ambient data shown in the Fig. S5b. (c) Methane concentration enhancements under the 10×baseline scenario. (d) Methane concentration enhancements under EDGAR emission field.

Fig. S6 shows the bias between observed and simulated methane concentration enhancements on the 0.01°×0.01° grid under different specifications of methane emissions in four blocks. For the four shale gas blocks, the simulation driven by the baseline shale gas emissions shows a better agreement with observations than that driven by a 10× higher shale gas emission. This result demonstrates that the ambient observations support our inference of a low methane emission based on stationary downwind measurements.

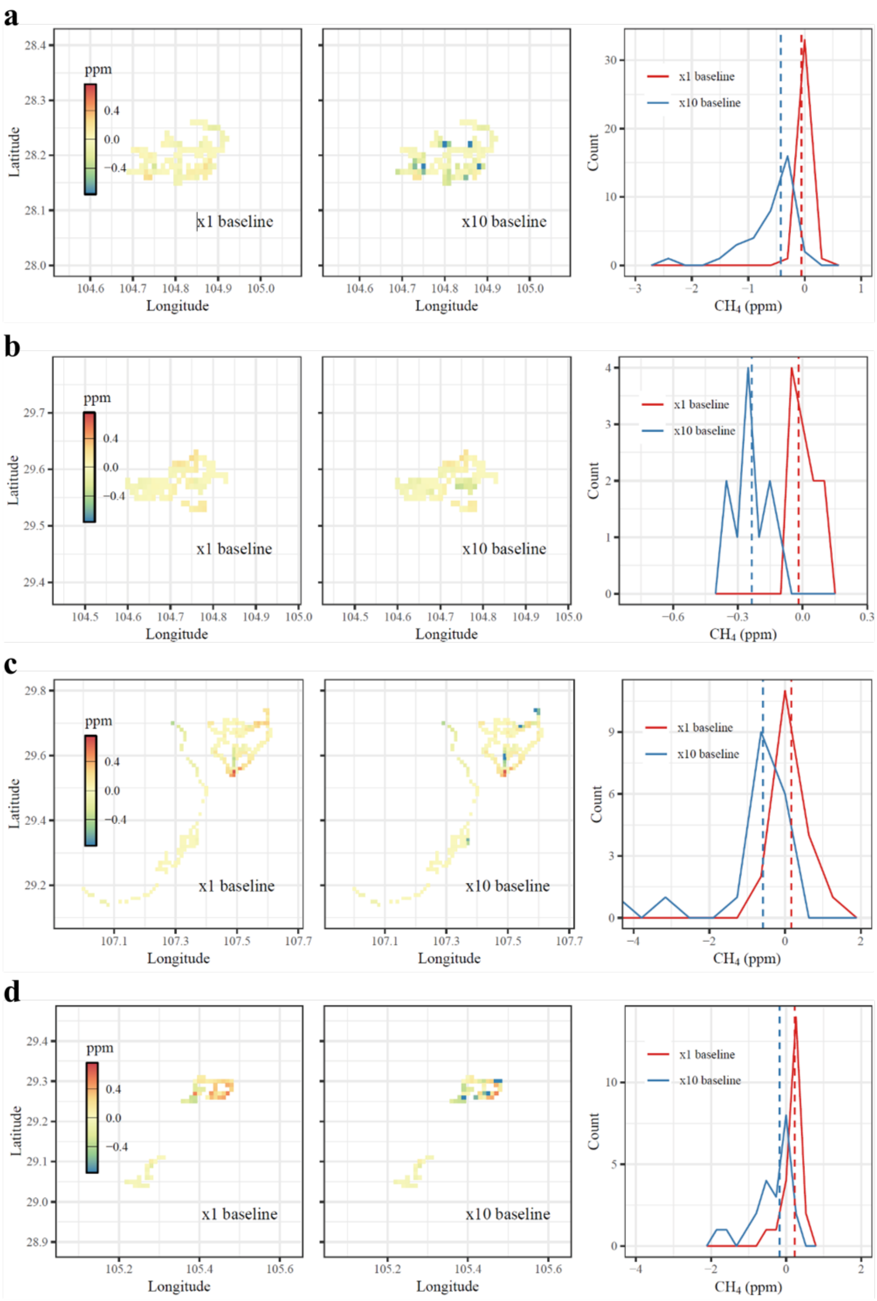

Figure S6. The differences between observed and simulated methane concentration enhancements in four blocks (Changning: a, Weiyuan: b, Fuling: c, and Luzhou: d). Bias between observed and simulated methane concentration enhancements under (Left) baseline methane emissions derived from downwind near-source measurements and 10 times enhanced methane emissions (Middle) in four blocks. Distribution of observation-simulation bias under the above two emission scenarios in grid cells that are sensitive to shale gas emissions. Vertical dashed lines show the median of the distributions (Right).

S7. Emission rate data

Table S2. Emission rate data from well pads in the four blocks after screening. We conducted stationary downwind monitoring in the Changning, Weiyuan, Fuling, and Luzhou blocks to obtain emission rate data from the wells. After further screening, the data that meets the quality standards is as follows, with the valid data counts for Changning, Weiyuan, Fuling, and Luzhou being 27, 22, 26, and 15, respectively.

Changning		Weiyuan		Fuling		Luzhou		Super emission	
well site No.	Emission rate (kg/h)	well site No.	Emission rate (kg/h)	well site No.	Emission rate (kg/h)	well site No.	Emission rate (kg/h)	well site No.	Emissio n rate (kg/h)
1	0.003	1	0.002	1	0.004	1	0.007	1	4.077
2	0.009	2	0.003	2	0.014	2	0.010	2	4.306
3	0.009	3	0.009	3	0.028	3	0.019	3	6.041
4	0.017	4	0.010	4	0.030	4	0.070	4	7.305
5	0.025	5	0.011	5	0.037	5	0.074	5	34.634
6	0.025	6	0.014	6	0.039	6	0.118	6	1.078
7	0.025	7	0.020	7	0.052	7	0.161	7	6.300
8	0.028	8	0.027	8	0.059	8	0.255	8	2.736
9	0.030	9	0.029	9	0.077	9	0.377	9	67.552
10	0.032	10	0.048	10	0.081			10	2.075
11	0.077	11	0.087	11	0.082			11	4.969
12	0.088	12	0.099	12	0.093			12	8.170
13	0.120	13	0.159	13	0.108			13	24.305
14	0.130	14	0.241	14	0.109			14	36.248
15	0.131	15	0.503	15	0.111			15	84.084
16	0.180	16	0.549	16	0.132			16	113.638
17	0.201	17	0.553	17	0.168				
18	0.210	18	0.677	18	0.176				
19	0.290	19	0.685	19	0.177				
20	0.324	20	1.069	20	0.232				
21	0.465			21	0.248				
22	2.642			22	0.628				
				23	0.888				
				24	1.528				

Table S3. Emission rate data that has not passed data quality screening

Changning		Weiyuan		Fuling		Luzhou	
well site No.	Emissions rate (kg/h)	well site No.	Emissions rate (kg/h)	well site No.	Emissions rate (kg/h)	well site No.	Emissions rate (kg/h)
1	0.003	1	0.008	1	0.008	1	0.012
2	0.008	2	0.013	2	0.010	2	0.017
3	0.011	3	0.017	3	0.054	3	0.018
4	0.017	4	0.027	4	0.208	4	0.026
5	0.018	5	0.030	5	0.840	5	0.029
6	0.018	6	0.049	6	1.555	6	0.031
7	0.048	7	0.073	7	4.797	7	0.045
8	0.056	8	0.733	8	16.62	8	0.046
9	0.630					9	0.074
10	0.747						

Supplemental Reference

(1) Pataki, D. E.; Ehleringer, J. R.; Flanagan, L. B.; Yakir, D.; Bowling, D. R.; Still, C. J.; Buchmann, N.; Kaplan, J. O.; Berry, J. A. The Application and Interpretation of Keeling Plots in Terrestrial Carbon Cycle Research. *Global Biogeochemical Cycles* **2003**, *17* (1), 2001GB001850. https://doi.org/10.1029/2001GB001850.

(2) Moreira, M.; Sternberg, L.; Martinelli, L.; Victoria, R.; Barbosa, E.; Bonates, L.; Nepstad, D. Contribution of Transpiration to Forest Ambient Vapour Based on Isotopic Measurements. *Global Change Biology* **1997**, *3* (5), 439–450. https://doi.org/10.1046/j.1365-2486.1997.00082.x.
